# Surgical results of the resection of spinal meningioma with the inner layer of dura more than 10 years after surgery

**DOI:** 10.1038/s41598-021-83712-0

**Published:** 2021-02-18

**Authors:** Hiroyuki Tominaga, Ichiro Kawamura, Kosei Ijiri, Kazunori Yone, Noboru Taniguchi

**Affiliations:** 1 Department of Orthopaedic Surgery, Graduate School of Medical and Dental Sciences, Kagoshima University, 8-35-1 Sakuragaoka, Kagoshima, 890-8520 Japan; 2 Kirishima Orthopaedic Surgery, Kirishima, Japan

**Keywords:** Neurological disorders, Outcomes research

## Abstract

Most spinal meningiomas arise from the thoracic dura in middle-aged and elderly women. Simpson grade 1 resection is recommended to avoid recurrence. For ventral and ventrolateral tumors, reconstruction after total dural resection is difficult, and spinal fluid leakage is likely. To overcome this concern, Saito et al*.* developed the technique of resecting the tumor with the inner dural layer, preserving the outer dural layer. Although meningioma rarely recurs, the recurrence period is approximately 8 years postoperatively. No studies have evaluated long-term (> 10-year) outcomes of the Saito method. Here, we report 10 cases of the Saito method with > 10-year follow-up and compare outcomes with those of other standard approaches. Twenty-nine pathology-confirmed meningioma patients underwent surgery in our department, ten with the Saito method. We investigated resection method (dura mater treatment), pathological type, and recurrence and compared pre- and postoperative clinical findings. The median follow-up was 132 months. Recurrence occurred after Simpson grades 3 and 4 resection. Simpson grades 1, 2, and the Saito method resulted in no recurrence. Neurological symptoms improved in all patients at final follow-up. This is the first report of long-term outcomes of the Saito method. The method achieved good neurological improvement with no recurrence in > 10-year follow-up.

## Introduction

Spinal cord meningioma is an intradural, extramedullary tumor that occurs most frequently at the thoracic level in middle-aged and elderly women. Simpson grade 1 resection (macroscopically complete tumor resection with removal of affected dura and abnormal bone) of spinal meningioma is considered desirable to prevent recurrence long after surgery^1^. In the case of ventral and ventrolateral tumors, reconstruction after total dural resection is difficult, and spinal fluid leakage is likely to occur even after reconstruction with thoracolumbar fascia or artificial dura. To overcome this concern, Saito et al*.* developed an alternative technique in which the tumor is resected with the inner dural layer alone, preserving the outer dural layer^2^. Although meningioma rarely recurs, the reported mean recurrence period is approximately 8 years^3,4^. However, the long-term (> 10-year) risk of recurrence and other side-effects of the Saito method remain unclear. Here, we report the 10-year outcomes of 10 cases in which the Saito method was used and compare the risks of this method with those of other standard approaches.

## Methods

Forty-five patients diagnosed pathologically with meningioma underwent surgery in our department from 1992 to 2010; 29 of these were tracked over this period (follow-up rate: 64.4%). We investigated age, sex, resection method (dura mater treatment), tumor location, pathological type, and recurrence.

**Figure 1:**
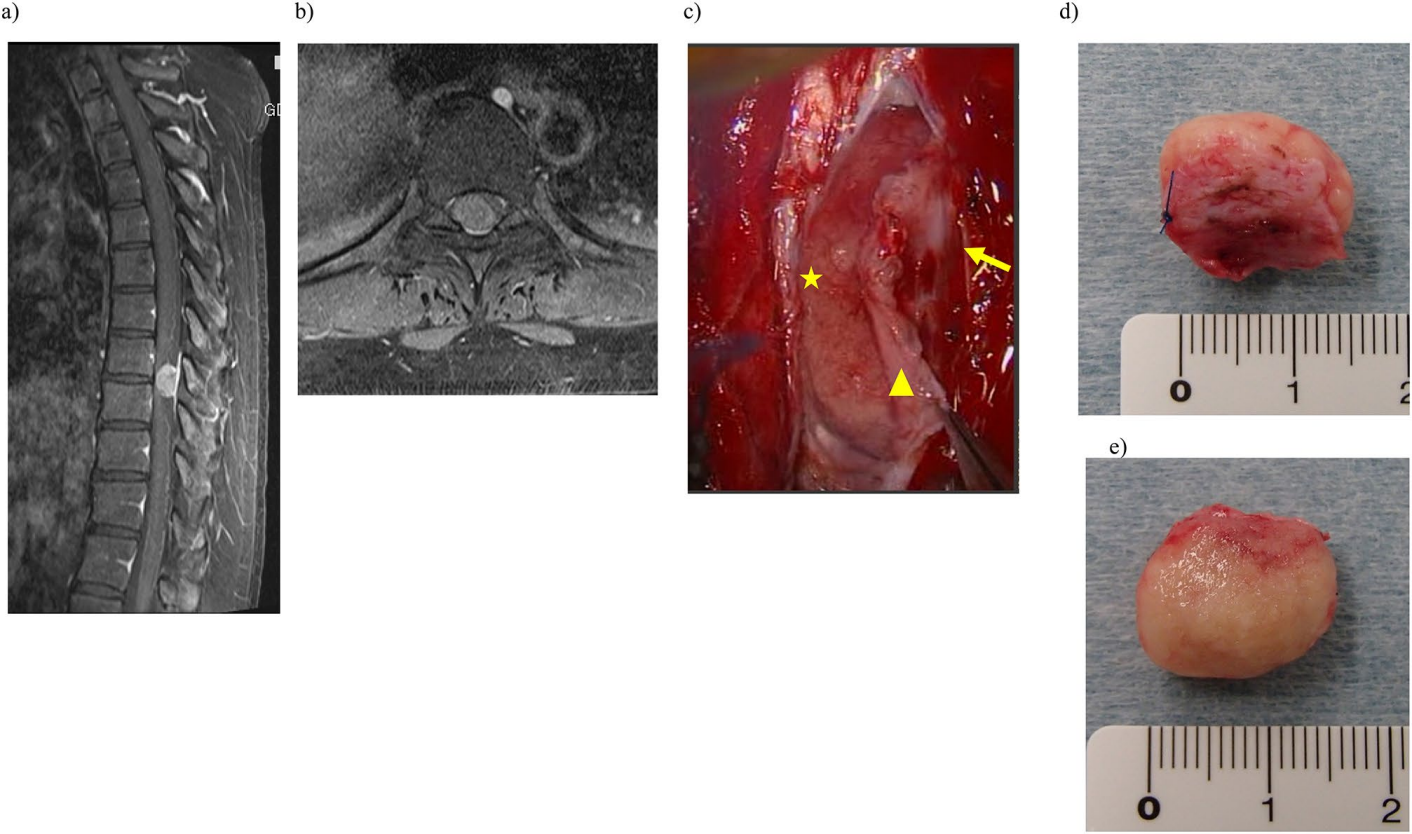
Resection of meningioma at T8/9 level in a 44-year-old woman. T1-weighted magnetic resonance image (MRI) with gadolinium enhancement. (**a**) Sagittal and (**b**) axial views. We measured the maximum tumor length on MRI. (**c**) Resection of meningioma with inner dural layer (triangle), outer dural layer (arrow), and tumor (star). Excised specimen showing (**d**) dural side and (**e**) ventral side. We confirmed attachment of inner dural layer to the tumor.

Since 2003, we have usually performed tumor resection by removing the tumor with the inner dural layer as a single mass after separating the inner and outer layers of the dura mater (Fig. 1)^2^. In an alternative procedure, we remove the inner layer of the dura with adhesions after tumor resection.

During tumor resection, we used a Cavitron ultrasonic surgical aspirator to avoid traction on the spinal cord. We have used ultrasound to determine tumor location since 2003. We determined the location of dural attachment with preoperative magnetic resonance imaging (MRI) and surgical findings^5^. We measured the maximum tumor length on MRI (Fig. 1). Radiological assessments were performed by two spine surgeons who were instructors accredited by the Japanese Society for Spine Surgery and Related Research. The Japanese Orthopaedic Association score was used to clinically evaluate the patients. We measured the rate of symptom improvement by using the methods of a previous report^6^.

Parameters were compared with the Wilcoxon test and Fisher’s exact test. *P* < 0.05 was considered statistically significant. The JMP software program (version 15; SAS Institute, Cary, NC, USA) was used for statistical analysis. The study protocol was approved by the local ethics committee of Kagoshima University, and all patients provided written informed consent. All methods were carried out in accordance with relevant guidelines and regulations.

## Results

**Figure 2:**
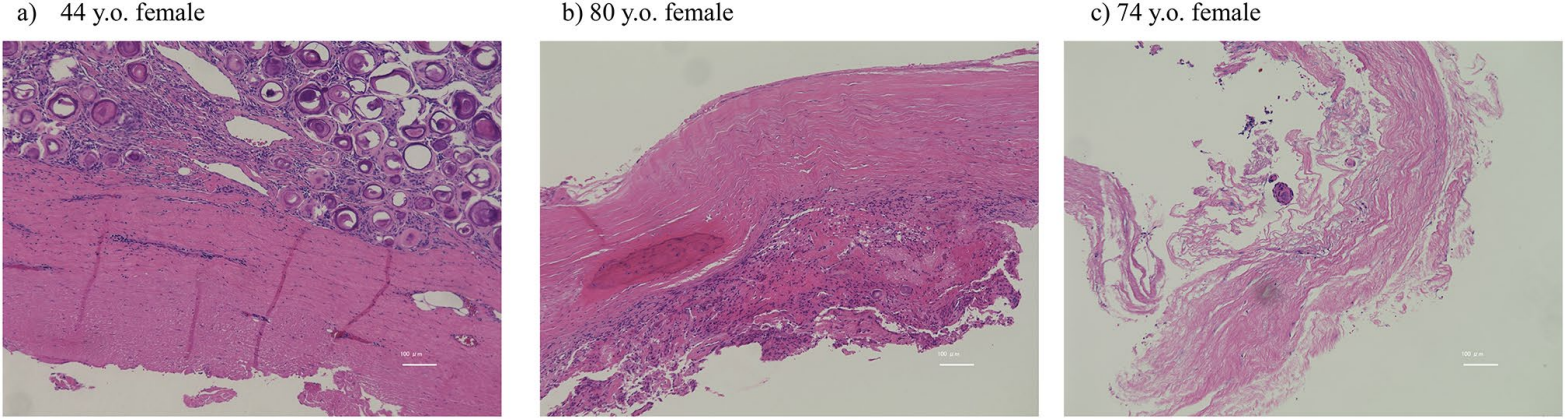
Pathology. Photomicrograph of resected tumor stained with hematoxylin and eosin. (**a**) Forty-four-year-old woman; psammomatous type. (**b**) Eighty-year-old woman; meningothelial type. (**c**) Seventy-four-year-old woman; meningothelial type. We removed the tumor with the inner layer of dura as a single mass in Case a and removed the adhesed inner layer of dura after tumor resection in Cases b and c. No infiltration of tumor cells in the dura mater was seen in Case a or b, but tumor infiltration was confirmed in Case c.

The median patient age was 64 years (range, 49.0–70.5 years), and 22 of the patients were women. The median follow-up period was 132 months (range, 120.0–160.5 months) (Table 1). The pathological subtype was meningothelial in 13 cases, psammomatous in 13 cases, transitional in 2 cases, and fibrous in 1 case (Table 1). The method of resection ^7^ was Simpson 1 in 5 cases, resection of meningioma with the inner dural layer in 10 cases, Simpson 2 in 3 cases, Simpson 3 in 10 cases, and Simpson 4 in 1 case (Table 2). In patients with ventral or ventrolateral tumors, we generally resected the meningioma with the inner layer of dura. Recurrence was confirmed in three cases of Simpson 3 or 4 resection (Table 2). The median recurrence period was 95 months (Table 3). The median thoracic JOA score before Saito method surgery was 5.2; the median score improved significantly to 10.0 at the final follow up (*P* < 0.001). We performed reoperation in all cases of recurring meningioma because of worsening neurological symptoms. There was no recurrence among patients who underwent Simpson 1 or 2 resection or resection of the inner dural layer. We examined pathological samples in some cases and detected tumor cells infiltrating the inner dural layer (Fig. 2). Tumor recurrence was observed in patients who underwent Simpson 3 or 4 resection; there was a statistically significant difference in resection type (Simpson 3/4 versus other) between the recurrent group and the non-recurrent group (*P* < 0.05).

**Table 1:** Patients’ characteristics.

Number of patients	29
Age, years	64.0 (49.0–70.5)
Sex, female	22 (75.9%)
Follow-up duration, months	132.0 (120.0–160.5)
Tumor location	
Cervical	5
Thoracic	23
Lumbar	1
Pathological type	
Psammomatous	13
Meningothelial	13
Transitional	2
Fibrous	1

All patients were World Health Organization (WHO) grade 1.

Data are presented as number, number (%), or median (interquartile range).

**Table 2:** Resection method.

Resection method	Location of dural attachment to spinal meningioma				Recurrence	Total
	Dorsal	Dorsolateral	Ventrolateral	Ventral		
Simpson 1	2	2	1	0	0	5
Simpson 2	0	1	1	1	0	3
Simpson 3	1	2	7	0	2	10
Simpson 4	0	0	0	1	1	1
Tumor resection with inner layer of dura	2	0	8	0	0	10

Values indicate number of patients.

**Table 3:** Characteristics of patients with recurrence of meningioma.

Age, years	Sex	Follow-up, months	Location	Simpson grade	Pathological subtype	MIB-1
23	Female	113	Dorsal	3	Meningothelial	−
69	Female	64	Ventral	4	Psammomatous	3%
47	Female	95	Ventrolateral	3	Psammomatous	2%

The median duration until recurrence was 95 months. MIB-1 index: Ki-67 scoring.

Postoperative neurological symptoms improved compared with preoperative symptoms in all cases. There were no statistically significant differences in tumor size or symptom improvement rates between patients who underwent Simpson 1 resection and those who underwent resection of meningioma with the inner dural layer (Table 4).

**Table 4:** Tumor size and patients’ clinical results according to Japanese Orthopaedic Association score.

	Simpson 1	Tumor resection with inner layer of dura	*P*-value
Maximum length of tumor, mm	16.1 (15.1–17.5)	17.3 (16.2–18.9)	0.18
JOA improvement rate, %	81.8 (75–92.3)	77.3 (57.5–93.2)	0.54

JOA improvement rate (%) = ([post JOAs] − [pre JOAs] / [total JOAs] – [pre JOAs]) × 100.

JOAs: Japanese Orthopaedic Association score.

Data are presented as the median (interquartile range) and were analyzed with the Wilcoxon test.

## Discussion

To our knowledge, this is the first report evaluating the long-term results (> 10 years) of internal dural resection based on the Saito method^2^. Saito et al*.* reported that the spinal dura can be divided into two layers and recommended resection of tumor with the inner dural layer only. This technique decreases the risk of postoperative cerebrospinal fluid fistula, compared with complete resection of the dura mater. Simpson grade 1 resection, in which the entire dura is resected at the tumor attachment site, is considered desirable to prevent recurrence of spinal meningioma long after surgery; Simpson grade 2, in which the inner layer of dura is coagulated at the tumor attachment, is considered inadequate^1^. In the case of ventral and ventrolateral tumors, reconstruction after total dural resection is difficult, and cerebrospinal fluid leakage is likely to occur, even with the use of artificial dura^3^. Cerebrospinal fluid leakage may be less likely with Simpson grade 2 resection because the dura is preserved^3^; however, it can be difficult to identify the dural site of tumor attachment. Therefore, we chose to use Saito’s method, in which the tumor attachment is resected along with the inner dural layer, leaving the outer layer intact.

Previous studies have reported that dural cauterization after meningioma removal (Simpson 2) was successful ^8,9^ and that dural lining resection was effective^2^. Others have reported no recurrence in cases of total dural resection (Simpson 1)^1,10^. One study found no difference in recurrence rates between Simpson 1 and 2 resection^3^. However, all cases in that study were observed for less than 10 years; the mean radiographic follow-up period before tumor recurrence was 95.4 months.

The largest spinal meningioma cohort study involved 483 cases ^4^ and demonstrated that Simpson grade 3 resection was a risk factor for recurrence. The authors reported a recurrence rate of 4.6%, with a mean radiographic follow-up period of 94 months before tumor recurrence. In the present study, we also found recurrence in cases of Simpson 3 or 4 resection. Another study showed that ventrally located spinal meningiomas had a recurrence rate of 0% after Simpson grade 2 resection^11^. That study had a mean follow-up of 40.6 months, which may have been too short to accurately evaluate recurrence. In the present study, the mean radiographic follow-up period until tumor recurrence was 95 months, which was similar to that in a previous study^3^. When evaluating meningioma recurrence, it is necessary to observe patients for more than 10 years after surgery.

To our knowledge, only five reports, including this study, have included more than 10 years of radiographic follow-up after spinal meningioma resection^1,8,12,13^. Dorsal and dorsolateral meningiomas are significantly more likely to be treated with Simpson grade 1 resection, whereas ventral and ventrolateral meningiomas are significantly more likely to be treated with Simpson grade 2 resection^3^. The complication rate associated with Simpson grade 1 resection is significantly higher than that associated with Simpson grade 2^3^. Studies have shown that artificial dura mater reconstruction leads to adhesion arachnoiditis^14,15^, and with Simpson 1 resection, postoperative cerebrospinal leakage can worsen symptoms^16^.

In this study, we generally used Saito’s method in ventrolateral cases in which dural treatment was difficult, and no spinal fluid leakage or recurrence was observed. Dura mater reconstruction was not required after internal dural resection in any case because of postoperative cerebrospinal fluid leakage.

Maiuri et al. reported that the proliferation index, Ki-67, and arachnoid invasion may be risk factors for recurrence of spinal meningioma^13^. The authors reported no significant differences in recurrence rates after Simpson 1 versus Simpson 2 resection. However, one case showed tumor infiltration in the epidural layer on pathological examination, and the tumor was then excised. Meningioma tumor cells in the dura have been reported with Simpson 1 resection^17^. In the present study, tumor cells were present in the resected inner layer of dura. Although no tumor recurrence was observed in more than 10 years of follow-up, ongoing careful observation is necessary.

This study has several limitations. First, it was retrospective. Second, a small number of cases were evaluated. Because of the small number of cases, it is difficult to compare recurrence rates after the Saito method with those after Simpson 1 or 2 resection. It is necessary to confirm the superiority of long-term results with studies involving more cases at other institutions.

However, the Saito method achieved good neurological improvement with no cases of recurrence for at least 10 years. It is significant that we have reported 10 cases of the Saito method with more than 10 years of follow-up, which had not been reported until now. This technique might be a good addition to the surgical options for spinal meningioma.

## Conclusions

This is the first report of long-term results (> 10 years for internal dural resection) of the Saito method. The Saito method resulted in good neurological improvement with no recurrence for at least 10 years. This technique might be a good addition to the surgical options for spinal meningioma.

## Data Availability

The datasets generated during and/or analyzed during the current study are available from the corresponding author on reasonable request.
